# Supplementary material for: Acoustic wave response to groove arrays in model ears
Source: PLoS One. 2021 Nov 29;16(11):e0260020. doi: 10.1371/journal.pone.0260020 (PMC8629259; doi:10.1371/journal.pone.0260020)
Supplement: Attachment — Submitted filename: Response to Reviewers.docx [file pone.0260020.s005.docx]

Re:

PONE-D-21-14020

Groove arrays in the ears of mammals and owls enhance acoustic information.

PLOS ONE

Re: Rebuttal Letter

Dear PlosOne editor(s) and reviewers,

Thank you for taking the time to review and comment on this study. The comments have helped to strengthen the quality and clarify many of the complex concepts for the readers. We have addressed each comment and hopefully have resolved all major concerns. In response to reviewer questions, we went through the document and made appropriate modifications as requested.

The reviewers’ questions/comments have been bolded to keep them separate from ours.

**5. Review Comments to the Author

Please use the space provided to explain your answers to the questions above. You may also include additional comments for the author, including concerns about dual publication, research ethics, or publication ethics. (Please upload your review as an attachment if it exceeds 20,000 characters)**

**Reviewer #1: This is an interesting experimental investigation into the role of grooves patterns that can be found in bat ears. However, if I understand the description of the methods and the results correctly, the comparisons between grooved and smoothed shapes were done using only single acoustic measurement for each shape. In practice, acoustic measurements and spectrum estimates based on them are subject to a very high level of variability. It is hence absolutely essential that the authors add multiple repetitions to their measurements and investigate which of the observed differences are statistically significant. Without such an analysis, it is impossible to draw any conclusions from the obtained results.**

It is stated in the manuscript in that both the 40kHz continuous tone and the prey generated sounds have five recordings each for each distance that those tests involved. The data presented are from averaging those five recordings. As requested by Reviewer #1 we have included statistical analysis of data for the 40 kHz continuous tone recordings. The statistical analysis for the 40 kHz continuous tone data and the difference graphs for the prey generated comparisons are being submitted in the supplementary information (S1).

The data from the prey generated sounds recordings for each model is a broadband signal that is not in a format that can easily be statistically compared in a meaningful way. In the manuscript, we used a representative difference graph as our method to show the comparative relationship of how and where two broadband signals can vary. At your request, we have now included the difference graphs for the smooth vs the array models for each distance in the supplemental data.

**In addition, I believe that it is essential for the authors to remove all mentions of "acoustic information" from their hypotheses since there is nothing in their work/analysis that could be seen as measuring information.**

We removed all mentions of “acoustic information” and replaced with wording like “signal response” or something similar depending on the context.

**Finally, the authors should comment on how the studied frequency range compares to the frequency bands known to be utilized by bats and owls.**

A discussion on hearing ranges for animals with groove arrays was added to the Discussion section (with appropriate referencing).

**Here some minor issues I have come across:

l. 47: There seems to be no benefit to using the term "polyphonic" here, which afaik means independent melodies, not just multiple
frequencies in music .**

Removed “polyphonic” replaced with “wavefronts containing multiple frequencies” throughout

**l. 82: Would say "phase relationships" to avoid confusion with medical use of phasic (i.e., short-term).**

Inserted your suggestion

**l. 83: Use "broadband" instead of "polyphonic".**

Changed as suggested

**l. 123: It is not informative to say that the pinna models were based on "discussions with acoustic specialists", instead the specialists should be thanked in the acknowledgments.**

Made appropriate adjustments to text

**l. 134: The "z" in "kHz" is not capitalized.**

corrected

**l. 150: More information about the prey noise should be given, e.g.,
bandwidth, spectral shape (flat, lowpass, bandpass characteristics).**

Modified text to direct readers to a reference where it is fully described.

**l. 196 - 198: It appears that no repetitions of the expriments.**

In line 198 it clearly states that 5 repetitions were made for the 40 kHz continuous tone and prey generated sounds tests.

**l. 340: Capitalize "B" in "dB".**

changed

**l. 359: Remove comma after "Kuc".**

Corrected

**Reviewer #2: This paper presents that the groove arrays on the ear model increase the signal intensity compared to the smooth model. It provides fundamental study for many further open questions. There are a few concerns that could be addressed to improve the quality of the paper.

1. This paper mainly investigates the effect of groove array on the general ear model instead of the animal’s ear. I’d recommend that the Short Title is more appropriate than the Full Title.**

Swapped as stated

**2. A setup figure (or a sketch of the emitter, ball, sandpaper discs, etc) shows how those three sound sources are generated will help the reader to understand.**

A request is submitted to the Editor to add this figure. This addition will surpass the number of figures recommended by the journal. All other images are deemed essential. In fact, this image did exist during the formulation of the manuscript and was removed due to the allowable limit.

**3. Including example spectrograms of array model and smooth model in Fig 6 could help the reader to understand how you get the dB difference plots.**

Thank you for the suggestion. We know that this is a complicated concept, but we are concerned that including additional images within figure 6 may introduce confusion for an already complex concept. Plus, the spectrographs of prey generated sounds are broadband graphs that are not that helpful to readily depict the differences visually (see inserted images below). You can see slight differences in intensity, but the important details exist in the comparable values for specific frequencies. Thus, we had to figure out a method to efficiently compare the differences between any two broadband signals. We overcame the variability inherent to all acoustic signals by conducting multiple tests and averaging them and then learned that subtracting those averaged values against those we wished to compare. This technique made it easy to expose where and by how much differences exist and were then able to graph those differences using the difference graph technique. This technique enabled us to compare any two values generated by all models. We hope the explanatory text accompanied by the figure will suffice.

**4. Please make sure all figures have x-axis and y-axis labels/units and suitable font sizes. In fig 8, the time duration (1000 ms) in the x-axis should be consistent for all subplots.**

We have altered the text fonts and units as requested so that they meet PlosOne criteria. The samples portrayed in Fig 8 were cut from actual recordings, so the X axis labeling can slightly vary based on where the cut was taken even though we tried to take them from about the same area. However, because each image represents the echo from six passes of a rotating ball and the ball was traveling at a consistent speed in all recordings, the recordings all have six pulses and are approximately the same length. To address your concerns a note was added in the caption indicating that all plots represent approximately 1000 ms.

**5. In line 50 “Huygens-Fresnell principle” should be “Huygens-Fresnel principle”.
In line 250 “...intense. in signal strength...”.
In line 338 “db” should be “dB”.**

Corrected. Thank you.

**6. In line 131, is there any specific reason to choose the ear model dimensions to be 28 mm X 44 mm?**

The designs were based on several factors including array groove widths. The models were designed to accommodate all variations of groove numbers and widths we were interested in testing which included ones with sets of 20 grooves if they would fit. Some bats have 20 grooves. The widest groove width we needed has a measurement of 1.715mm that matches the wavelength found in a 200 kHz signal. That requires a length of 34.3 mm to fit 20 grooves. The full length of the model (44mm) includes the basal chamber features. The width is roughly ½ the length and is based on proportional features often found with bats and other animals that have large ears and are heavily reliant on acoustic information. The model designs likely do not come close to the efficiency of real animal ears that have been tuned by evolution, but the important thing is that the comparison tests are consistent among the models. To make accurate comparisons, the shape and size of the models must be able to accommodate the wavelengths of interest and the tests must be (and were) conducted in an identical format using uniformly shaped and sized models in the same orientation. We hope this sufficiently addresses this question.

**7. Is the statement in lines 308-309 based on the results from fig 6? If so, it is different from the statement of lines
236-237.**

Good catch! They were both correct but indistinctly if not awkwardly worded. One is about the greatest difference in amplitude where the other is proportionally the strongest. To reduce the potential for confusion, we removed the first statement from lines 236-237 and replaced the lines in the Discussion section with two sentences that read:

The single strongest difference in amplitude occurred between the smooth model and the 1.715mm 10 groove model at a distance of 40cm resulting in a signal that was 32 dB more intense which was 34% stronger (1.715mm 10 groove= -57.7 dB; smooth=-89.8 dB). The strongest comparative change in proportion occurred between the smooth and the 3mm width 10 groove model at a distance of 10cm resulting in a signal that was only 4 dB more intense but was 54% stronger (3mm10g=-3.5 dB; smooth=-7.5 dB).

**8. The statement in line 314: “The influence of the arrays appears to diminish as the distance increases with one sound source but not another.” Replace the “one sound source, another” with the specific name of the sound source toavoid ambiguity.**

Added specifics

**9. In the “Discussion” section, the author mentioned “traditional diffraction grating studies” many times and compared their results with it. References are needed to the “traditional diffraction grating studies”.**

Added references where appropriate.

**10. Did the prey sound test involve 10 models and 5 distances as well? If so, any further statistical analysis could be
put into the supplementary material. Currently, only one fig (fig 7) shows one condition.**

Yes an entire series was conducted matching what was done for all models at all distances in the 40 kHz continuous tone and the prey generated sounds tests. We have added the requested results in the supplementary data.

The data from the prey generated sounds recordings for each model is a broadband signal that is not in a format that can easily be statistically compared in a meaningful way. In the manuscript, we used a representative difference graph as our method to show the comparative relationship of how and where two broadband signals can vary. At your request, we have now included the difference graphs for the smooth vs the array models for each distance in the supplemental data.

**11. In lines 337-338: “This study demonstrates that the presence of arrays appears to be able to enhance the signal
strength across the spectral envelope, but the effective distance may vary according to the sound source.”
The “sound source” here indicates 3 different types of sound sources that are continually mentioned in the paper or
different frequency sounds from the "40kHz sound source"? It should be stated more clearly.**

We reworded selected text to clarify the responses attributed to specific sound sources. We hope we succeeded.

**Reviewer #3: There are two major issues with this paper.
1. The ridges do much better than the pinna without ridges, particularly the increase in gain. Could there be a problem with that pinna that evolution has solved using thin flexible skin?**

We do not discount the concept that the skin can have the ability to sense acoustic signals in ways we do not yet comprehend. It is a simple rule of physics that waves respond to structure. Our tests show that this is the case. A relatively consistent property among animals heavily reliant on acoustics (i.e., nocturnal) is the presence of a groove array, especially those that fly or operate in a 3-dimensional world (flying, gliding and arboreal). In fact, almost all bats have arrays, even those that do not echolocate (i.e., flying foxes) as do many other mammalian orders (see our first paper, citation 4) and we now know they exist in owls as well. We also know that animals have evolved a well-developed hearing system and can navigate in complex structure at high speeds in near or absolute darkness. We are assuming that by morphologically tapping these acoustical properties, evolution has assisted their ability to exploit nocturnal resources. However, there is still the possibility that the skin can also contribute to how the animal takes advantage of acoustic energy, but that is a different study.

**2. More important than 1: The paper gives quantitative and qualitative results that indicate the effect of the ridges, but little analysis or explanation on how they would benefit a bat. The experiments and ridge designs were too exhaustive in scope. This reviewer would have liked to see one or two signals with the best ridge design, in the author's opinion, to demonstrate the benefit of the ridges, beyond that the ridges modify the detected signals.**

This is a complicated topic that is not easily exposed. The primary goal of the study was to determine if the ridges modify the acoustic signal. For the tests we conducted, we found that certain characteristics do it better than others and we feel it is important to understand those differences. A cursory review of images of the ears of bats and other animals reveals that there are many variations of the ridge arrays in nature. We want to know which of these variations are important, hence the many variations we chose to test. The many differences undoubtedly have meaning to the animals, and we are hoping to stimulate further research, so we feel it is important to share at least a partial range of what we have learned so far.

We also know that there are different ways acoustic signals are encountered or made by an animal either passively from sounds generated by the environment or actively through vocalizations, hence the multiple sound sources.

This paper is an introductory view of a subject that has not been thoroughly studied. We feel that it is important to share the broader view of what we found with this study. We include a short list of questions in hopes that future studies will begin to focus on specific issues.
