# Supplementary material for: Acoustic wave response to groove arrays in model ears
Source: PLoS One. 2021 Nov 29;16(11):e0260020. doi: 10.1371/journal.pone.0260020 (PMC8629259; doi:10.1371/journal.pone.0260020)
Supplement: Attachment — Submitted filename: Response to reviewers.docx [file pone.0260020.s006.docx]

October 17, 2021

RE: PlosOne Response to Reviewers

PONE-D-21-14020R1

Acoustic wave response to groove arrays in model ears.

PLOS ONE

Response to comments received Tue, Sep 28 at 8:32 AM

Dear Reviewers and PlosOne Editors,

Our responses to the September 28, 2021 review comments are italicized below.

Reviewer #1: “The revision address all my concerns with the exception of the variability in the measurements. The manuscript should clarify whether 5 measurements were made for each of the 5 different distances, or just one measurement was made per distance. In the former case, error bars (e.g., max and min differences found) should be included in Fig. 6 to show the variability of the measurements. If only 1 measurement per distance has been made been made, additional measurements should be done to assess the variability in these experimental results.”

*Reviewer #1 has offered valid concerns and requested information to strengthen the integrity of the data and the study results.*

*We address the issue of variation using the 5 replicate recordings for both the 40 kHz continuous tone and the manufactured prey recordings described at lines 198, 211, 216, 232, 238.*

*To account for the requested information on variability, we added the error bars to Fig 6 in support of the 40 kHz continuous tone data as requested and for the prey generated recordings, we added supporting statistical information in the supplementary sheets (S2).*

*Although the 126 kHz echo pulses were useful to visually demonstrate how the models uniquely influence the signal, we had no intentions of using that recording statistically and did not do multiple replicates to account for variability, so we removed it and any reference to it from the paper and modified the text accordingly.*

*We added explanatory paragraphs in the Analysis and Results sections to reflect what was done to address variability and where to find relevant data.*

*We made minor edits throughout the text where necessary to improve readability.*

*We hope this satisfies the reviewers concerns,*

*Brian*
